# Interplay between Endometriosis and Pregnancy in a Mouse Model

**DOI:** 10.1371/journal.pone.0124900

**Published:** 2015-04-27

**Authors:** Mariela Andrea Bilotas, Carla Noemí Olivares, Analía Gabriela Ricci, Juan Ignacio Baston, Tatiana Soledad Bengochea, Gabriela Fabiana Meresman, Rosa Inés Barañao

**Affiliations:** Instituto de Biología y Medicina Experimental (IBYME)—Consejo Nacional de Investigaciones Científicas y Técnicas (CONICET), Buenos Aires, Argentina; Michigan State University, UNITED STATES

## Abstract

**Objectives:**

To evaluate the effect of endometriosis on fertility and the levels of the IL-2 and IFN-γ in the peritoneal fluid in a mouse model; to evaluate the effect of pregnancy on endometriotic lesion growth, apoptosis and cell proliferation.

**Study Design:**

Two month old C57BL/6 female mice underwent either a surgical procedure to induce endometriosis or a sham surgery. Four weeks after surgery mice were mated and sacrificed at day 18 of pregnancy. Number of implantation sites, fetuses and fetal weight were recorded. Endometriotic lesions were counted, measured, excised and fixed. Apoptosis and cell proliferation were evaluated in lesions by TUNEL and immunohistochemistry for PCNA respectively. Levels of IL-2 and IFN-γ were assessed by ELISA in the peritoneal fluid.

**Results:**

Pregnancy rate (*i*.*e*. pregnant mice/N) decreased in mice with endometriosis. However there were no significant differences in resorption rate, litter size and pup weight between groups. IFN-γ augmented in endometriosis mice independently of pregnancy outcome. Additionally IFN-γ increased in pregnant endometriosis mice compared to pregnant sham animals. While IFN-γ increased in non pregnant versus pregnant mice in the sham group, IL-2 was increased in non pregnant mice in the endometriosis group. The size of endometriotic lesions increased in pregnant mice while apoptosis increased in the stroma and cell proliferation decreased in the epithelium of these lesions. Additionally, leukocyte infiltration, necrosis and decidualization were increased in the same lesions.

**Conclusions:**

Pregnancy rate is reduced in this mouse model of endometriosis. Levels of IL-2 are increased in the peritoneal fluid of mice with endometriosis suggesting a role of this cytokine in infertility related to this disease. The size of endometriotic lesions is increased in pregnant mice; however pregnancy has a beneficial effect on lesions by decreasing cell proliferation and by increasing apoptosis, decidualization and necrosis.

## Introduction

Endometriosis is a chronic gynecological disorder associated with pelvic pain and infertility. It is characterized by the presence of endometrial glands and stroma outside the uterine cavity and is estimated to occur in up to 10% of women of reproductive age [1,2].

Although the association between endometriosis and infertility is well supported throughout the literature, a definite cause-effect relationship has not been found. Different studies suggest that 20% to 50% of infertile women suffer from endometriosis and that 30% to 50% of women with endometriosis are sub-fertile or infertile [3,4,5,6].

According to literature the presence of endometriotic lesions produces a cytokine imbalance in the peritoneal environment [7,8]. Infertility in women with endometriosis is believed to be related to these alterations because the pelvic cavity, uterus, fallopian tubes and ovaries are bathed in the peritoneal fluid [6]. High levels of IFN-γ have been seen in spontaneous abortion in women, additionally IL-2, TNF-α or IFN-γ injected intraperitoneally (i.p.) increase abortion rates in mice [9,10,11]. Furthermore, higher concentration of IL-2 has been observed in the peritoneal fluid of infertile patients with endometriosis compared to controls [12]

In addition, it is widely accepted that endometriosis symptoms tend to improve during natural pregnancy [13,14]. Moreover, it has been proposed that pregnancy has a suppressive effect on endometriotic lesion growth and recurrence [14]. McArthur *et al*. have observed a reduction in non-ovarian endometriotic lesion size during pregnancy, however they have also found some lesions of augmented size [15]. Moreover, a recent study showed that the size of ovarian endometriotic cysts decreases during pregnancy in only 52% of the cases analyzed; whereas there is no change in 28%, and it increases in 20% [16]. Also several reports have shown serious complications of endometriosis during pregnancy, such as perforations of the appendix and sigmoid colon, rupture of the uterus affected by endometriosis and hemoperitoneum and spontaneous bleeding from blood vessels secondary to endometriosis [17,18,19,20,21,22,23]. These complications are infrequent but they are potentially life threatening and are increasing because more patients with endometriosis can achieve pregnancy through the use of assisted reproduction techniques [21].

Based on the reviewed data, the objective of the present work was to evaluate the effect of endometriosis on fertility and the levels of the IL-2 and IFN-γ in the peritoneal fluid in a mouse model. In addition we evaluated the effect of pregnancy on endometriotic lesion growth, apoptosis and cell proliferation.

## Materials and Methods

### Animals

C57BL/6 two month old female mice were used in this study. Animals were kept in ventilated cages (Tecniplast, Buguggiate, Italy) with free access to water and food in a 12 hour light/dark cycle. All experimental procedures were performed according to NIH Guidelines for the Care and Use of Laboratory Animals and approved by the Ethics and Research Committee from IBYME, Buenos Aires, Argentina.

### Surgery

**Fig 1:**
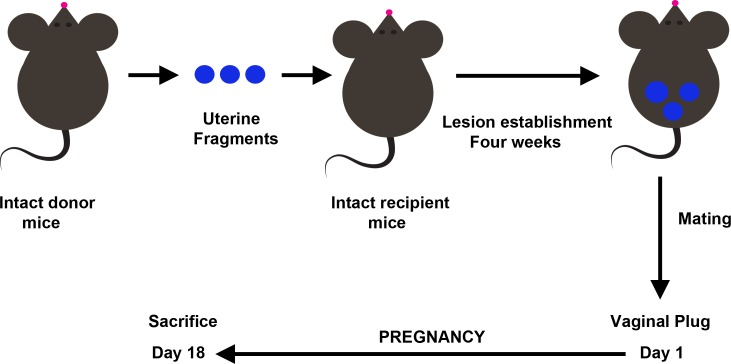
Mouse syngeneic model of endometriosis and experimental design.

Endometriotic lesions were experimentally induced by syngeneic transplantation of uterine tissue onto the bowel mesentery as previously described (Fig 1) [24,25,26]. Briefly, donor animals were sacrificed by cervical dislocation and both uterine horns were removed and placed into warm medium (DMEM F12, Gibco, Paisley, United Kingdom). The uterine horns were opened longitudinally and then cut into square pieces measuring approximately 4 mm^2^. Recipient syngeneic mice were placed under general anesthesia with an i.p. injection of ketamine (100 mg/kg) and xylazine (10 mg/kg) and a mid-ventral incision was made to expose the bowels. Three equal pieces of one uterine horn from a donor mouse were sutured onto the colonic mesentery with the endometrial layer facing the bowel serosa. A single suture (Supralon 6–0, Ethicon, Somerville, New Jersey) was used for each piece of uterine tissue. The abdomen was then closed using the same suture material.

Because surgery itself can have effects on peritoneal cytokines, we have chosen sham animals as the control group. These animals underwent the same surgical procedure as the endometriosis group with the difference that suture was performed onto the colonic mesentery without uterine tissue. In this way, we were able to distinguish the effects of the presence of endometriosis in the peritoneal cavity from any effect that could be a consequence of the surgery itself.

All efforts were made to minimize suffering.

### Experimental design

All mice were undergoing normal estrous cycle as we confirmed by the vaginal cytology method [27]. There were no differences in the vaginal cytology between sham and endometriosis groups. Four weeks after surgery 20 sham females and 22 females with surgically induced endometriosis were mated irrespective of the day of the estrous cycle. Groups of two female mice were placed in cages with a male of proven fertility until the visualization of a vaginal plug (up to 12 days; *i*.*e*. 3 reproductive cycles). The presence of vaginal plug was considered day 1 of pregnancy and these females were separated and placed in new cages. Mice that failed to get pregnant after vaginal plug visualization were included in the non pregnant group (4 sham mice and 9 mice with endometriosis). Animals were euthanized by CO_2_ at day 18 post mating (day 18 of pregnancy). The pregnancy rate was defined as the ratio of the number of pregnant mice to N.

Endometriosis model and experimental design are summarized in Fig 1.

### Necropsy

After sacrifice, mice were immediately fixed in the supine position on a platform and the abdomen was opened. Immediately, peritoneal fluid was collected by rinsing the abdominal cavity of all mice with 1.5 ml of saline. Afterwards, it was centrifuged at 300xg for 5 minutes to remove cells. In pregnant mice, number of implantation sites, fetuses and fetal weight were recorded, and a gross external evaluation of the pups for malformations was performed. In mice with surgically induced endometriosis, lesions were identified, counted and measured in two perpendicular diameters using a caliper. The volume of each lesion was calculated by the following formula: V = (4/3)πr^2^R (where r and R are the radiuses and r < R) [24]. Lesions were then dissected away from intestinal tissue, peritoneum and adhesions and fixed in 4% buffered formaldehyde for two days for histological analysis. Formalin-fixed specimens of ectopic tissue were paraffin-embedded, cut into 5 μm sections and stained with hematoxylin and eosin. Sections were examined microscopically for the presence of histological hallmarks of endometriosis.

### Apoptosis detection system

For apoptosis quantification, ectopic tissue sections were processed for terminal deoxynucleotidyl transferase (TdT)-mediated dUTP-fluorescein nick-end labeling (TUNEL) staining using the “In Situ Cell Death POD” kit (Roche, Basel, Switzerland). Sections were treated according to the manufacturer's instructions. Briefly, sections were deparaffinized in xylene, rehydrated through graded alcohols and permeabilized with 20 μg/ml Proteinase K (Gibco, Grand Island, NY, USA). Endogenous peroxidase was inactivated by coating the samples with 3% H_2_O_2_. Sections were rinsed with PBS, and then immersed 60 min in TdT buffer at 37°C. Sections were incubated 30 min with the anti-fluorescein peroxidase antibody, followed by the peroxidase substrate DAB. Finally, sections were counterstained with hematoxylin. Slices of female rodent mammary gland obtained 3–5 days after weaning of pups were used as a positive control. As a negative control, a number of tissue samples were subjected to treatment without TdT.

The percentage of TUNEL positive cells was established using a standard light microscope by two independent observers at 400X and any nuclear brown stain was considered as positive. At least 600 epithelial and 600 stromal cells were counted per mouse considering all lesions. Independently of the number of cells at least 10 representative fields were counted per mouse.

### Evaluation of cell proliferation by immunohistochemical staining for Proliferating cell nuclear antigen (PCNA)

PCNA was studied from the same paraffin-embedded samples on serial sections using an immunohistochemical method. Briefly, sections were deparaffinized in xylene and rehydrated through graded alcohols, followed by microwaving in 0.01M sodium citrate buffer for antigen retrieval. Endogenous peroxidase was blocked by treatment with 3% H_2_O_2_ for 10 min and non-specific binding was blocked by incubation with normal rabbit serum for 60 min. Tissue sections were incubated for 60 min with anti-mouse PCNA rabbit polyclonal antibody (1:400, FL-261, Santa Cruz Biotechnology Inc., Santa Cruz, CA, USA) at room temperature. After that, sections were treated for 30 min with a secondary biotin-conjugated anti-rabbit antibody followed by incubation with peroxidise-conjugated streptavidin (LSAB+ System, Dako). Binding was visualized by incubating sections with DAB, and lightly counterstaining with hematoxylin, prior to permanent mounting. As a negative control, immunoglobulin of the same immunoglobulin class and concentration as the primary antibody was used. PCNA positive cells were identified by the presence of brown nuclear reactivity and any nuclear staining was regarded as positive.

PCNA, also called cyclin, is a 36-KD auxiliary protein of DNA polymerase-delta, that has been found to be a useful marker in immunocytochemical studies of cell proliferation because its expression correlates with the proliferative state of the cell [28].

The percentage of PCNA positive cells was established using a standard light microscope by two independent observers at 400X and any nuclear brown stain was considered as positive. At least 600 epithelial and 600 stromal cells were counted per mouse considering all lesions. Independently of the number of cells at least 10 representative fields were counted per mouse.

### Quantification of IL-2 and IFN-γ

Peritoneal fluid IL-2 and IFN-γ concentrations were assessed in mice using a mouse Th1/Th2 ELISA Ready-Set-Go! kit (eBioscience, San Diego, CA, USA). The sensitivity levels for IL-2 and IFN-γ assays were 2 pg/ml and 15 pg/ml respectively. All samples were assayed in duplicate.

### Statistics

Statistical analysis was performed using Statistica software (StatSoft Inc,Tulsa, USA). Statistical comparisons were carried out by Chi-square test, Student “t” test and two-way ANOVA test followed by Tukey Honest significant difference (HSD) test for unequal sample size. Regardless of the statistical test, only a p value ≤ 0.05 was considered significant. Results are expressed as mean ± SEM.

## Results

### Effect of endometriosis on fertility

Because endometriosis is characterized by infertility we assessed pregnancy rate, resorption rate, litter size, pup weight and number of days lapsed until the presence of vaginal plug in a mouse model of endometriosis.

Pregnancy rate was significantly lower in females with endometriosis compared to sham mice (Table 1). However there were no significant differences in resorption rate, litter size, pup weight and number of days lapsed until the presence of vaginal plug between the group of mice with endometriosis and the sham group (Table 1).

**Table 1:** Effect of endometriosis on fertility.

	Sham	Endometriosis	P value
	N = 20	N = 22	
Presence of vaginal plug (days) ^**a**^	3.1±0.5	2.7±0.6	NS
Pregnancy rate (%) ^b^	80	56	P<0.05 ^c^
Litter size (N) ^a^	8.1±0.4	7.2±0.8	NS
Pup weight (g) ^a^	1.0±0.1	0.9±0.1	NS
Resorption rate (%) ^b^	2	7	NS

^a^ Statistical comparisons were performed by Student “t” test

^b^ Statistical comparisons were performed by Chi-square test

^c^ Sham vs. Endometriosis

### Effect of endometriosis and pregnancy on peritoneal IL-2 and IFN-γ

It is known that the presence of endometriotic lesions alters peritoneal environment. Since high levels of IL-2 and IFN-**γ** are related to infertility and reproductive organs are bathed in the peritoneal fluid we evaluated the levels of these cytokines in pregnant and non pregnant mice with and without endometriosis.

**Fig 2:**
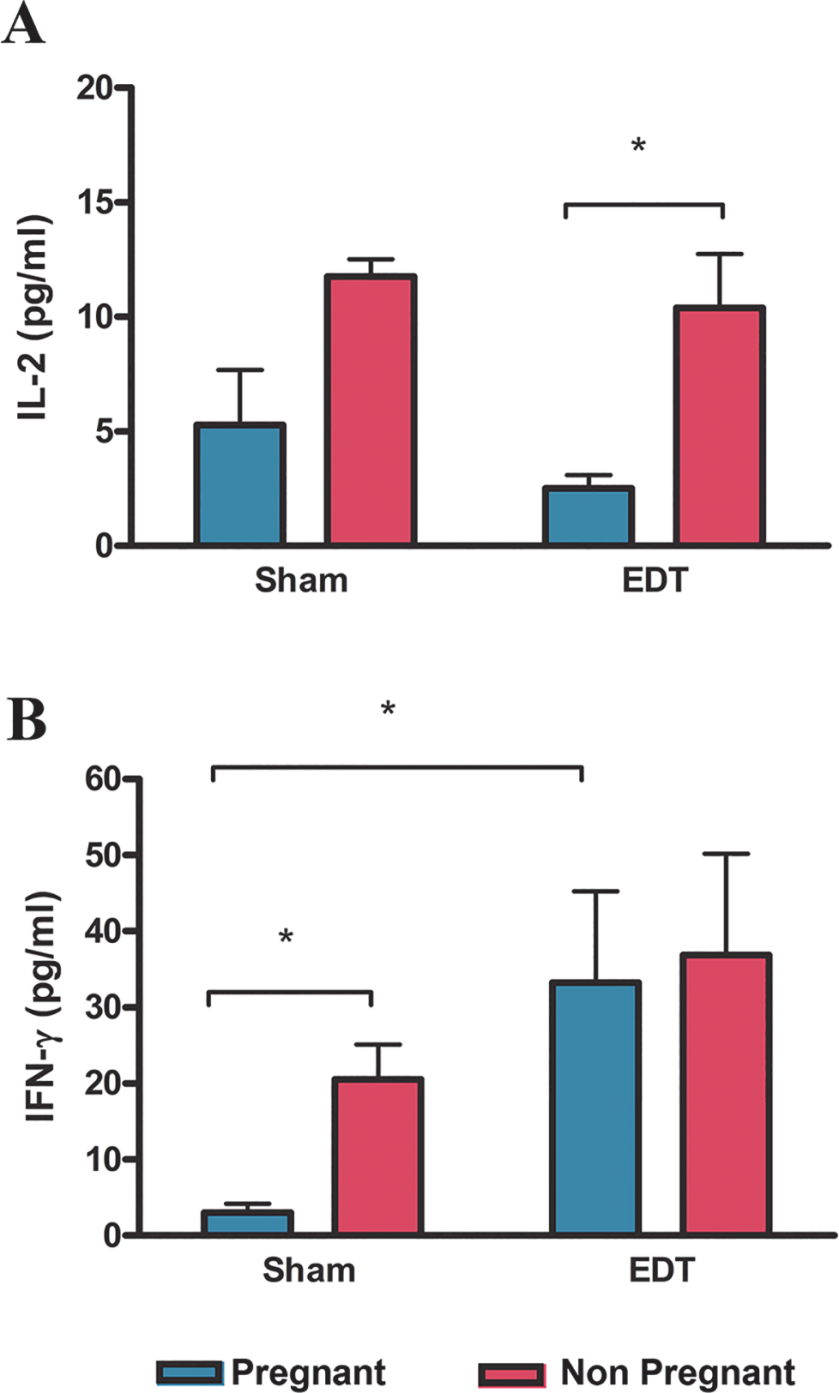
Effect of pregnancy and endometriosis on peritoneal IL-2 and IFN-γ. Mice with surgically induced endometriosis (EDT; N = 20, 11 pregnant) and sham animals (N = 18, 14 pregnant) were mated and sacrificed at day 18 of pregnancy. Levels of IL-2 (A), and IFN-γ (B) were assessed in the peritoneal fluid by ELISA. Statistical comparisons were performed by two way ANOVA test followed by Tukey HSD test for unequal sample size. * p<0.05

IL-2 was increased in the peritoneal fluid of non pregnant mice compared to the pregnant ones in the group with endometriosis (Fig 2A). Although peritoneal IL-2 levels are higher in non pregnant sham mice than in pregnant ones, these differences are not significant (Fig 2A).On the other hand, the levels of IFN-γ were elevated in the peritoneal fluid in non pregnant mice compared to the pregnant ones in the sham group (Fig 2B), while there were no differences in the endometriosis group (Fig 2B). In addition, IFN-γ was increased in pregnant mice with endometriosis versus sham pregnant mice (Fig 2B). Moreover, the levels of IFN-γ were higher in the peritoneal fluid from mice with endometriosis compared to sham mice independently of the pregnancy outcome (35.1±8.6 pg/ml vs. 12.0±3.6 pg/ml respectively; p<0.05). There were no differences either in IL-2 or in IFN-γ levels between non pregnant mice with and without endometriosis.

### Effect of pregnancy on endometriotic lesion growth and histology

It is accepted that pregnancy improves endometriosis symptoms however its effect on endometriotic lesions’ growth is variable; and serious complications of endometriosis during pregnancy has been reported. Therefore we decided to evaluate the effect of pregnancy on endometriotic lesion growth and histology in a mouse model.

**Fig 3:**
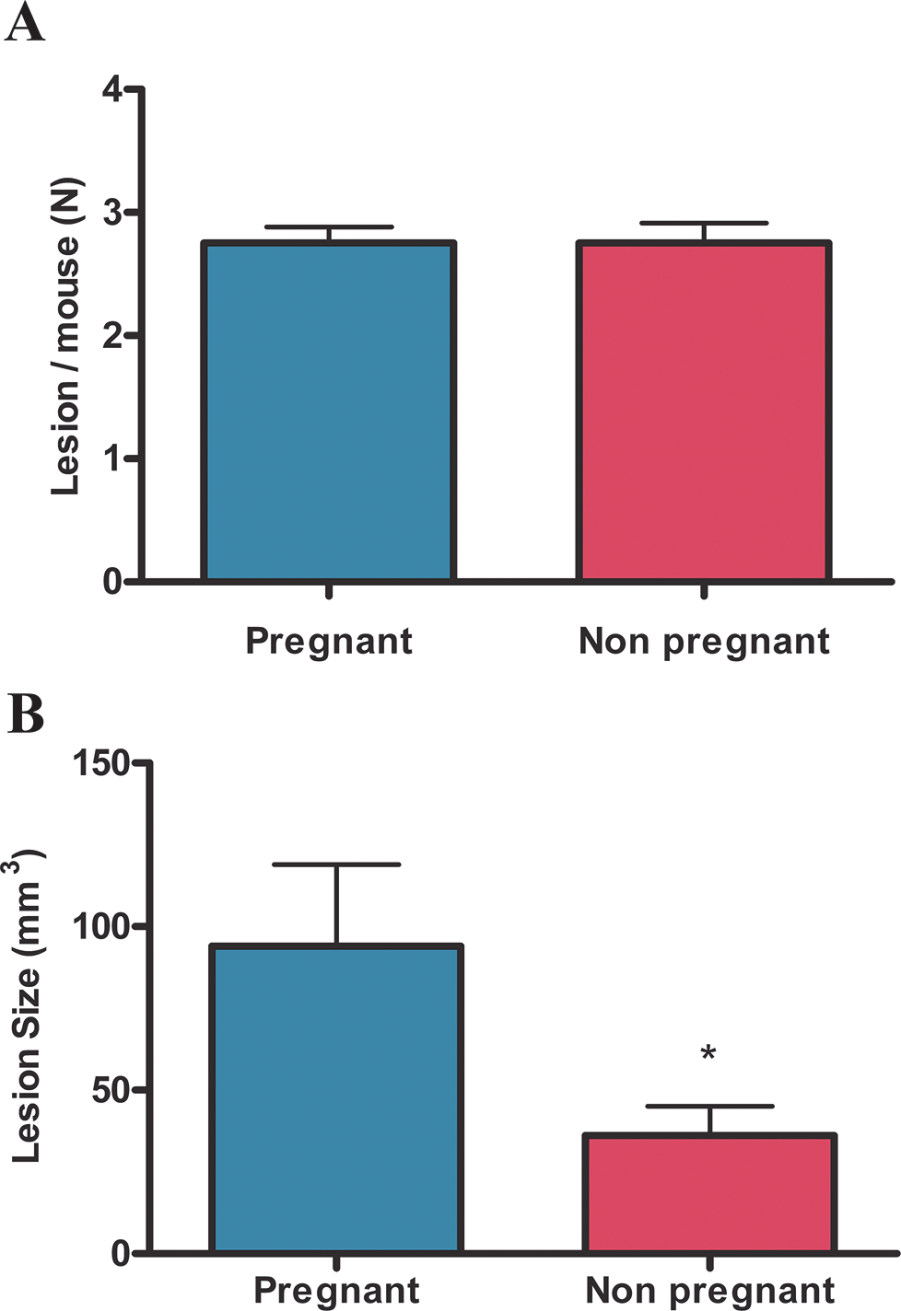
Effect of pregnancy on the number and size of endometriotic lesions. Mice with surgical induced endometriosis were mated and sacrificed at day 18 of pregnancy. The number of endometriotic lesions per mouse (A) and the size of endometriotic lesions (B) in pregnant (N = 11) and non pregnant (N = 9) mice were assessed. Statistical comparisons were performed by Student “t” test. * p<0.05 pregnant vs. non pregnant

All animals developed endometriotic lesions: 25% of the mice developed 2 lesions and 75% developed 3. There were no differences in the number of endometriotic lesions developed per mouse between pregnant and non pregnant mice (Fig 3A) however the size of endometriotic lesions was significantly larger in the pregnant group (Fig 3B).

**Fig 4:**
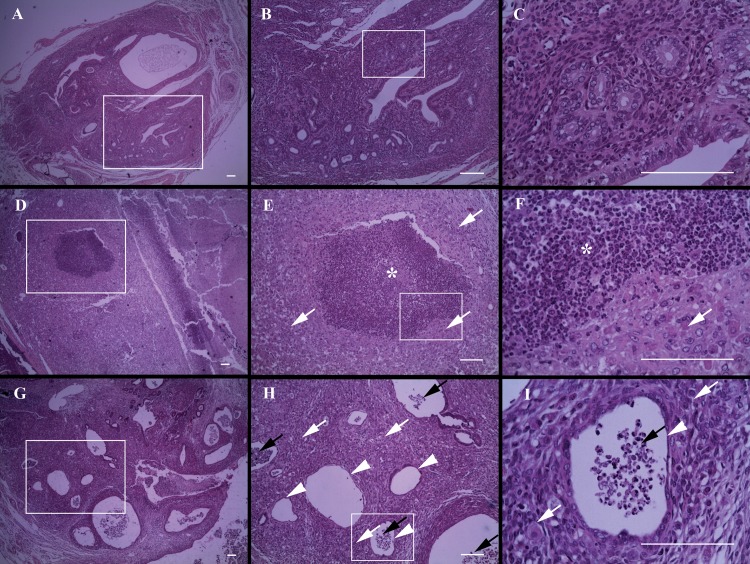
Effect of pregnancy on endometriotic lesion histology. Mice with surgical induced endometriosis were mated and sacrificed at day 18 of pregnancy. Micrographs show representative histological sections of endometriotic lesions from non pregnant (A-C) and pregnant mice (D-I). Asterisks show necrotic zones and black arrows infiltrated zones, both characterized by the presence of inflammatory cells. White arrows indicate a decidualized stroma, characterized by the presence of large polygonal cells with a pale pink cytoplasm and a large round or oval nucleus. Arrowheads show endometrial involution, recognized by the presence of a flattened-glandular-epithelium or the lack of one. The white boxes are shown at a higher magnification to the right. (A, D, G: 40X; B, E, H: 100X; C, F, I: 400X). Scale bar indicates 100 μm.

We then analyzed histological sections of endometriotic lesions stained with hematoxylin-eosin. The percentage of lesions that showed infiltration, necrosis, decidualization or endometrial involution was significantly higher in pregnant mice than in non pregnant ones (Table 2). We histologically corroborated that the observed enlargement of lesions in the pregnant group was associated with the presence of necrosis (Fig 4D–4F), leukocyte infiltration (Fig 4G–4I) and a decidualized stroma (Fig 4D–4I). Also, a high percentage of lesions from pregnant mice presented signs of endometrial involution showing a flattened or even an absent glandular epithelium (Fig 4G–4I). Conversely, endometriotic lesions from non pregnant mice showed normal glands and stroma (Fig 4A–4C).

**Table 2:** Effect of pregnancy on endometriotic lesions.

	Pregnant	Non Pregnant	P value
	N = 11	N = 9	
Leukocyte infiltration	82%	53%	P<0.05^a^
Necrosis	82%	26%	P = 0.0001^a^
Decidualization	79%	-	P<0.0001^a^
Endometrial Involution	93%	16%	P<0.0001^a^

*Note*: Statistical comparisons were performed by Chi-square test. Values are the percentage of lesions that showed signs of leukocyte infiltration, necrosis, decidualization or endometrial involution.

^a^ Pregnant vs. non pregnant

### Effect of pregnancy on endometriotic lesion cell proliferation and apoptosis

Although endometriotic lesions were larger in pregnant mice they showed histological markers of involution. Therefore we further investigated the status of endometriotic lesions concerning cell proliferation and apoptosis levels.

**Fig 5:**
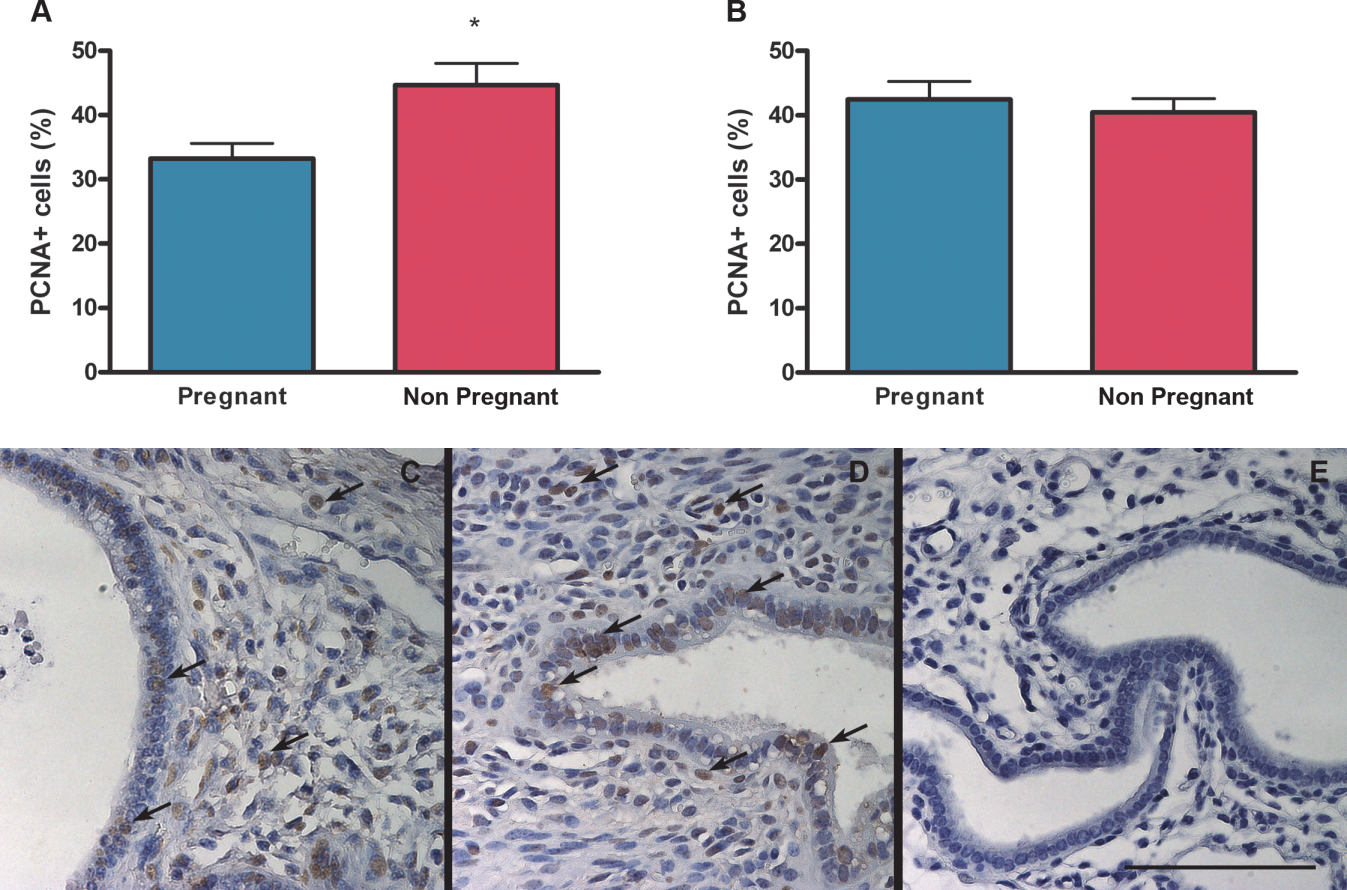
Effect of pregnancy on cell proliferation in endometriotic lesions. The percentage of proliferating cells was assessed by immunohistochemistry for PCNA in epithelial (A) and stromal (B) cells from pregnant (N = 11) and non pregnant (N = 9) mice. Micrographs (C-E) show representative histological sections of endometriotic lesions from pregnant (C) and non pregnant (D) mice. Arrows indicate marked cells. As a negative control, immunoglobulin of the same immunoglobulin class and concentration as the primary antibody was used (E). Scale bar indicates 100 μm. Statistical comparisons were performed by Student “t” test. * p<0.05 pregnant vs. non pregnant.

**Fig 6:**
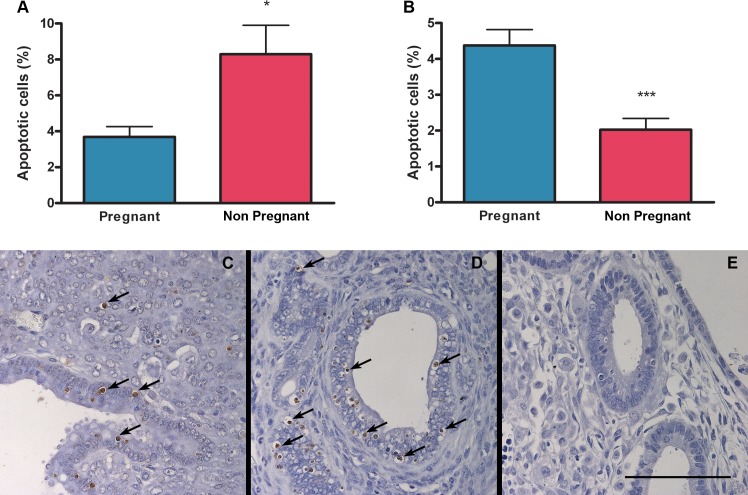
Effect of pregnancy on apoptosis in endometriotic lesions. The percentage of apoptotic cells was assessed by TUNEL in epithelial (A) and stromal (B) cells from pregnant (N = 11) and non pregnant (N = 9) mice. Micrographs (C-E) show representative histological sections of endometriotic lesions from pregnant (C) and non pregnant (D) mice. Arrows indicate marked cells. As a negative control, a tissue sample was subjected to treatment without TdT (E). Scale bar indicates 100 μm. Statistical comparisons were performed by Student “t” test. * p<0.05 pregnant vs. non pregnant; *** p<0.001 pregnant vs. non pregnant.

There were no differences in the percentage of proliferating cells in the stroma of endometriotic lesions between pregnant and non pregnant mice (Fig 5B–5D). However cell proliferation was decreased in the epithelium of endometriotic lesions from pregnant compared to non pregnant mice (Fig 5A, 5C and 5D). Additionally, the percentage of apoptotic cells increased in the stroma but decreased in the epithelium of endometriotic lesions from pregnant mice compared to non pregnant ones (Fig 6A–6D).

## Discussion

There are numerous studies that demonstrate an association between endometriosis and infertility [6,29]. The fecundity rate in patients with endometriosis is decreased compared to control women; furthermore, patients with mild endometriosis have a lower probability of getting pregnant than women with unexplained infertility [29,30].

The data from the present study demonstrate that pregnancy rate is decreased in mice with endometriosis compared to sham animals. Similar results have been shown in rats, but data seem to be contradictory in rabbits [31,32,33]. Despite the differences seen in pregnancy rate in this study, no significant changes were observed in resorption rate, litter size, pup weight or number of days lapsed until visualization of vaginal plug between the endometriosis and the sham group. These results are in agreement with the ones reported by Cummings *et al*. and Barragan *et al*. in mice and rats [31,34]. Nevertheless, other authors demonstrated a decrease in the number of pups in rats with endometriosis compared to the sham group [35]. Also Stilley *et al*. have found that the percentage of rats showing resorption sites is higher in those with endometriosis than in sham animals [36].

Several mechanisms have been proposed to explain the relationship between endometriosis and infertility [37]. Amongst them, it has been reported that the alterations observed in the peritoneal fluid in women with endometriosis contribute to the infertility related to this disease [6,37]. Hence, we analyzed the levels of IL-2 and IFN-γ in the peritoneal fluid of mice.

IL-2 was increased in the peritoneal fluid of non pregnant compared to pregnant mice with endometriosis but no statistical differences were seen within the sham group. Furthermore, the levels between the non pregnant animals do not show a statistical difference between groups and this particular result is in disagreement with the findings of Podgaec *et al*. who found higher levels of IL-2 in the peritoneal fluid of infertile patients with endometriosis compared to non pregnant controls [12]. Nevertheless, in agreement with Padgaec *et al*., our results suggest a role of IL-2 in endometriosis related infertility given that we found a statistically significant difference within the endometriosis group.

Conversely, IFN-γ is elevated in non pregnant mice compared to pregnant ones in the sham group but there were no differences in mice with endometriosis. In addition, IFN-γ is increased in peritoneal fluid of pregnant mice with endometriosis versus sham animals. Moreover, IFN-γ was elevated in the endometriosis versus the sham group independently of the pregnancy outcome. Taking these data altogether and because it is well known the detrimental role of IFN-γ on pregnancy, we cannot definitely discard a negative role of this cytokine on pregnancy in endometriosis [9,38,10]. However more studies are needed to further elucidate this.

There are several features of endometriosis that could explain the results obtained in this study. Women with endometriosis present impaired folliculogenesis, poor oocyte and embryo quality and defects in implantation [39,40]. Furthermore, peritoneal fluid from women with endometriosis alters sperm quality function [29,41]. Additionally, endometriosis in rats leads to ovulatory dysfunction and compromised oocyte and preimplantation embryo quality [36]. Although we have not assessed oocyte quality in any of the animals, we confirmed that they were all cycling normally.

It is important to notice that there are a few studies that have used the mouse model of endometriosis to evaluate infertility. Moreover, it has been proposed that the mouse model of endometriosis may appear more resistant than the rat model against effects of endometriosis on fertility [34]. Our work is valuable since it validates the use of the mouse model of endometriosis to study infertility. However, more studies are necessary to further investigate the factors responsible for the decrease in pregnancy rates observed in this work.

It is widely accepted that pregnancy has a suppressive effect on endometriotic lesions [42,43]. However, different studies showed that the size of endometriotic lesions not always decreases during pregnancy [16,15]. In the present work, no significant differences were observed in the number of endometriotic lesions developed per mouse between pregnant and non pregnant mice. Strikingly, we demonstrated that lesion size in pregnant mice is larger than in non pregnant ones. Opposite to our results, Cummings *et al*. showed that lesion size in pregnant mice with endometriosis was significantly smaller compared to non pregnant mice [34]. These discrepancies could be due to the fact that in our mice model, the uterine horns and the ovaries are intact while in the model used by Cummings *et al*. mice have only one uterine horn and ovary. Additionally, pregnancy has diverse effects on lesions in models of endometriosis in rat, baboon and cynomolgus monkey. In these models, pregnancy causes a complete regression of lesions, suppresses endometriosis only temporarily or has no effect [31,35,44,45,46].

Despite the fact that endometriotic lesions were larger in pregnant mice compared to non pregnant ones we propose a beneficial effect of pregnancy on endometriosis because apoptosis is increased in the stromal fraction and cell proliferation is decreased in epithelial cells of endometriotic lesions from pregnant compared to non pregnant mice. In addition, endometriotic implants in pregnant mice are decidualized, infiltrated with leukocytes and show signs of necrosis and endometrial involution. Although in this study epithelial glands present low levels of apoptosis our results suggest that they are in involution. Apoptosis and cell proliferation were evaluated in intact epithelium; however endometriotic lesions from pregnant mice show a high proportion of flattened or even absent glandular epithelium where it was not possible to evaluate these parameters.

In agreement with our results it has been shown that different synthetic progestins and progesterone itself increase apoptosis and have antiproliferative effects on rat endometrial cells and human endometrial and endometriotic stromal cells *in-vitro* [47,48,49,50,51]. In addition, large ovarian endometriotic lesions show decidualization, abscess formation and rupture during pregnancy in women [16]. Moreover, lesions treated with the progestin levonorgestrel present a poorly preserved epithelium or no epithelium at all in a rat model of endometriosis [52].

It is important to emphasize that there are few studies that have assessed the effect of pregnancy on endometriotic lesions, being this the first study that evaluates this effect on apoptosis or cell proliferation.

Taking into account these results we postulate that endometriotic lesions in pregnant mice are in regression even though they are larger. On the one hand, the decidualized stroma and the large areas with infiltration are the reasons for the enlargement of the endometriotic lesion [16,53]. On the other hand, apoptosis is increased in the stromal fraction and cell proliferation is decreased in the epithelial fraction of endometriotic lesions from pregnant mice. Furthermore, it has been postulated that decidualization and subsequent necrosis could explain the regression of endometriotic lesions during and after pregnancy [46,54].

In summary, this study demonstrates that there is a reduction in the pregnancy rate in this mouse model of endometriosis. Levels of IL-2 are increased in the peritoneal fluid of mice with endometriosis suggesting a role of this cytokine in infertility related to this disease. This work shows that the size of endometriotic lesions is increased in pregnant mice; however pregnancy has a beneficial effect on lesions by decreasing cell proliferation and by increasing apoptosis, decidualization and necrosis. Our work validates the use of the mouse model of endometriosis to study infertility and indicates that the size of endometriotic lesions during pregnancy would not always correlate with their activity.
